# The Kinase STK3 Interacts with the Viral Structural Protein VP1 and Inhibits Foot-and-Mouth Disease Virus Replication

**DOI:** 10.1155/2017/2481348

**Published:** 2017-10-31

**Authors:** Huisheng Liu, Qiao Xue, Qiaoying Zeng, Zixiang Zhu, Haixue Zheng

**Affiliations:** ^1^Laboratory of Veterinary Microbiology, College of Veterinary Medicine, Gansu Agricultural University, Lanzhou, China; ^2^State Key Laboratory of Veterinary Etiological Biology, National Foot and Mouth Diseases Reference Laboratory, Key Laboratory of Animal Virology of Ministry of Agriculture, Lanzhou Veterinary Research Institute, Chinese Academy of Agricultural Sciences, Lanzhou 730046, China

## Abstract

Foot-and-mouth disease virus (FMDV) is the etiological agent of FMD, which affects domestic and wild cloven-hoofed animals. The structural protein VP1 plays an important role in FMDV pathogenesis. However, the interacting partners of VP1 in host cells and the effects of these interactions in FMDV replication remain incompletely elucidated. Here, we identified a porcine cell protein, serine/threonine kinase 3 (STK3), which interacts with FMDV VP1 using the yeast two-hybrid system. The VP1-STK3 interaction was further confirmed by coimmunoprecipitation experiments in human embryonic kidney 293T and porcine kidney 15 (PK-15) cells. The carboxyl-terminal region (amino acids 180–214) of VP1 was essential for its interaction with STK3. The effects of overexpression and underexpressing of STK3 in PK-15 cells were assessed, and the results indicated that STK3 significantly inhibited FMDV replication. Our data expand the role of STK3 during viral infection, provide new information regarding the host cell kinases that are involved in viral replication, and identify potential targets for future antiviral strategies.

## 1. Background

Foot-and-mouth disease virus (FMDV), a positive-sense, single-stranded RNA virus, is the etiological agent of FMD, which affects domestic and wild cloven-hoofed animals, including cattle, pigs, sheep, goats, camelids, and deer [1, 2]. To date, seven serotypes (A, O, C, Asia, SAT1, SAT2, and SAT3) and numerous subtypes of FMDV have been identified, and no cross-protection has been reported among the different serotypes [3, 4]. The FMDV genome is approximately 8.5 kb in length, and it encodes a single polyprotein that is posttranslationally processed into four structural proteins (VP1, VP2, VP3, and VP4) and eight nonstructural proteins (L^pro^, 2A, 2B, 2C, 3A, 3B, 3C^pro^, and 3D) [5].

The contribution of each of these proteins to virulence during an infection of a natural host is not completely clear. To date, the proteins VP0, VP1, VP3, L^pro^, 2B, 3A, and 3C^pro^ have been reported to play roles in inhibiting or evading the host innate immune system [5–13]. In addition, some host cell proteins that interact with the FMDV proteins VP1, 2C, and 3A were identified by the yeast two-hybrid system [11, 14–16]. VP1, an important viral protein that plays an essential role in FMDV pathogenesis, carries the major neutralizing antigenic sites, and the VP1 gene has been used widely in epidemiological investigations of FMDV, vaccine development, and the establishment of diagnostic methods [17, 18].

To better understand the role of FMDV VP1 in viral replication and virulence, we aimed to identify new host cell proteins that interact with VP1 using the yeast two-hybrid system. Here, we report that VP1 binds to serine/threonine kinase 3 (STK3), a member of the mammalian STE20-like (MST) kinase family. The MST kinase family, which is related to the Hippo kinase in* Drosophila melanogaster*, consists of five related proteins: STK3 (also known as MST2), STK4 (also known as MST1), STK24 (also known as MST3), STK25 (also known as YSK1), and STK26 (also known as MST4) [19]. STK3 and STK4 are the key elements of the mammalian Hippo pathway, which influences cell proliferation, organ size, autophagy, apoptosis, and various stress responses [19–22]. Recent reports indicated that STK3 and STK4 play important roles in bacterial infections [20, 23, 24].

The VP1–STK3 interaction identified by the yeast two-hybrid system was further confirmed by coimmunoprecipitation experiments and an indirect immunofluorescence assay (IFA). Additionally, the amino acid residues in FMDV VP1 that mediate the interaction with STK3 were identified. Furthermore, overexpression of STK3 decreased FMDV replication within infected cells, while a knockdown of STK3 expression facilitated FMDV replication. Taken together, our data demonstrate an important antiviral role of STK3 in FMDV replication, and they expand the role of STK3.

## 2. Materials

### 2.1. Cells, Viruses, and Infection

Porcine kidney 15 (PK-15) cells and human embryonic kidney 293T (HEK293T) cells were cultured in Dulbecco's modified Eagle medium (Gibco) supplemented with 10% heat-inactivated fetal bovine serum (Gibco) and maintained at 37°C (5% CO_2_). The FMDV type O strain O/BY/CHA/2010 was used for viral infections as described previously [25].

### 2.2. Plasmids and Antibodies

The cDNA of porcine STK3 was amplified from PK-15 cells and cloned into the pcDNATM3.1/myc-His(−)A vector (Invitrogen, Carlsbad, CA, USA) to yield the Myc-tagged expression construct (Myc-STK3). A FLAG-tagged VP1 construct and a series of FLAG-tagged truncated VP1 constructs were prepared in our laboratory. All constructed plasmids were analyzed and verified by DNA sequencing. The commercial antibodies used in this study include an anti-Myc monoclonal antibody (Santa Cruz Biotechnology, Dallas, TX, USA), an anti-FLAG monoclonal antibody (Santa Cruz Biotechnology), an anti-FLAG polyclonal antibody (Sigma-Aldrich, St. Louis, MO, USA), an anti-STK3 polyclonal antibody (Abcam, Cambridge, MA, USA), and an anti-*β*-actin monoclonal antibody (Santa Cruz Biotechnology). An anti-VP1 polyclonal antibody was prepared in our laboratory [6].

### 2.3. Yeast Two-Hybrid Screen

A cDNA expression library was constructed using PK-15 cells. Total RNA was extracted using the RNeasy Extraction Kit (Qiagen, Valen-cia, CA, USA). Contaminating genomic DNA was removed by DNase treatment using Turbo DNA-free DNase (Ambion, Austin, TX, USA). Subsequently, RNA quality was assessed using RNA nanochips on a Bioanalyzer 2100 (Agilent Technologies, Santa Clara, CA, USA). GAL4-activation domain-cellular protein fusions were used as prey. The FMDV strain type O strain O/BY/CHA/2010 VP1, which was expressed as an amino-terminal fusion to the GAL4-DNA-binding domain, was used as the bait protein. Histidine and adenine were used for reporter genes for growth selection. The porcine library contained more than 3 × 10^6^ independent cDNA clones. For screening, a yeast strain expressing the VP1 protein was transformed with library plasmid DNA and selected on plates lacking tryptophan, leucine, histidine, and adenine. Once identified, positive library plasmids were recovered in* Escherichia coli (E. coli)* and sequenced to identify interacting cellular proteins. STK3 that was recovered from the library matched porcine STK3 (National Center for Biotechnology Information [NCBI] reference sequence GACC01000309.1).

### 2.4. Coimmunoprecipitation and Western Blot Analysis

HEK293T or PK-15 cells were seeded in 10-cm dishes, and monolayer cells were cotransfected with various plasmids. Then, the cells were collected and lysed, and proteins were immunoprecipitated as described previously [7]. The target proteins were resolved by sodium dodecyl sulfate-polyacrylamide gel electrophoresis and transferred onto an Immobilon-P membrane (EMD Millipore, Billerica, MA, USA). The membrane was blocked and then incubated with primary and secondary antibodies as described previously [26]. Antibody-antigen complexes were visualized by enhanced chemiluminescence detection reagents (Thermo Fisher Scientific, Waltham, MA, USA).

### 2.5. Indirect Immunofluorescence Microscopy

HEK293T cells were grown on Nunc™ glass bottom dishes and transfected with various plasmids using Lipofectamine 3000 (Invitrogen) according to the manufacturer's protocol. At 24 h after transfection (hpt), the cells were treated as described previously [6].

### 2.6. Knockdown of STK3 Using a Small Interfering RNA (siRNA)

The siRNAs used in this experiment were chemically synthesized by GenePharma (Beijing, China). The knockdown of endogenous STK3 in PK-15 cells was conducted by transfection with an STK3 siRNA. A nontargeting RNA (NC siRNA) was used as a negative control. siRNA transfection was performed using Lipofectamine 2000 (Invitrogen) according to the manufacturer's protocol. The target sequence for porcine STK3 was as follows:

  F: 5′-GCUGGAAAUAUUCUCCUUATT-3′,
  R: 5′-UAAGGAGAAUAUUUCCAGCTT-3′.

### 2.7. RNA Extraction and Quantitative Polymerase Chain Reaction (qPCR)

Total RNAs were extracted using TRIzol® reagent (Invitrogen). The isolated RNA was reverse transcribed to cDNA using the Moloney murine leukemia virus reverse transcriptase (Promega, Madison, WI, USA) and random hexamer primers (TaKaRa, Shiga, Japan). The transcriptional level of the mRNA was quantified by qPCR using SYBR Premix Ex Taq reagents (TaKaRa) in the Mx3005P qPCR System (Agilent Technologies). The glyceraldehyde-3-phosphate dehydrogenase (GAPDH) gene was used as an internal control. The qPCR primers used in this study are listed in Table 1. Relative fold changes of mRNA were calculated using the comparative cycle threshold (CT) (2^−ΔΔCT^) method [27]. All the experiments were repeated three times with similar results. The data represent results from one of the triplicate experiments.

### 2.8. Statistical Analysis

Statistical analysis was performed using SPSS Statistics for Windows, Version 17.0 (SPSS Inc., Chicago, IL, USA). Student's *t*-test was used for a comparison of three independent experiments. A *p* value < 0.05 was considered statistically significant (*∗*); A *p* value < 0.01 was considered statistically highly significant (*∗∗*).

## 3. Results

### 3.1. The FMDV Structural Protein VP1 Interacts with the Porcine Host Protein STK3

To date, the multiple functions of FMDV VP1 during viral infection remain unclear. To identify the host cellular proteins interacting with VP1 of FMDV, the FMDV VP1 was expressed as an amino-terminal fusion to the GAL4-DNA-binding domain. In the screen of a cDNA library of PK-15 cells using the yeast two-hybrid system, the plasmids in yeast were isolated, amplified in* E. coli* DH5*α*, and sequenced. Among them, one of these host proteins, identified as porcine STK3 (NCBI reference sequence GACC01000309.1), was selected for further immunoprecipitation analysis.

**Figure 1:**
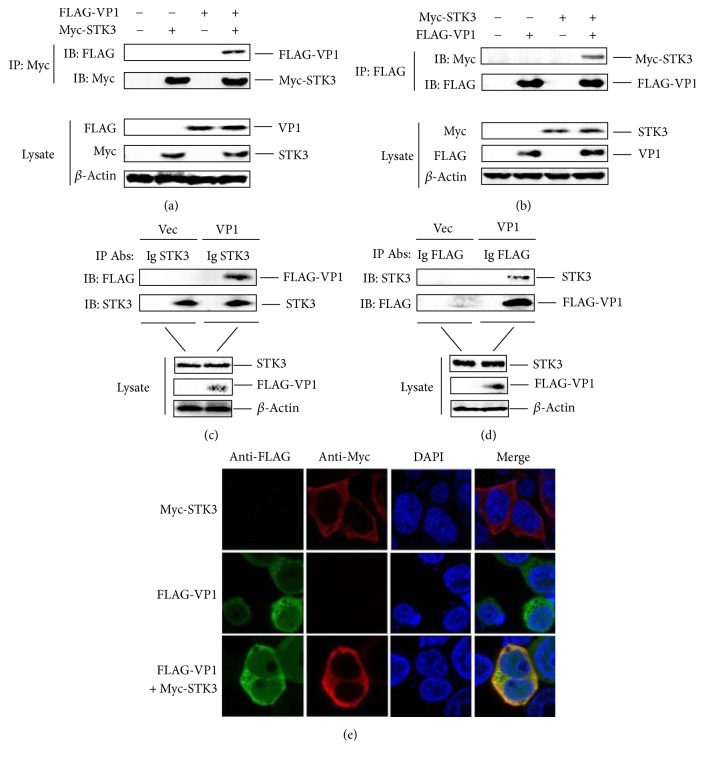
STK3 interacts with VP1. (a) HEK-293T cells were seeded in 10-cm dish, and the monolayer cells were transfected with 8 *μ*g of a Myc- STK3 expressing plasmid (+), 8 *μ*g of a FLAG-VP1 expressing plasmid (+), 8 *μ*g of an empty FLAG vector (−), or 8 *μ*g of an empty Myc vector (−). At 24 hpt, the cells were lysed, and the lysates were immunoprecipitated with mouse anti-Myc or mouse normal IgG antibodies and subjected to western blotting. The immunoprecipitated antibody-antigen complexes and whole-cell lysates were analyzed by immunoblotting using anti-FLAG, anti-Myc, or anti-*β*-actin antibodies. (b) Similar transfection and immunoprecipitation experiments were performed as described above. However, the lysates were immunoprecipitated with mouse anti-FLAG or mouse normal IgG antibodies and subjected to western blotting. (c) PK-15 cells were seeded in 10 cm dish, and the monolayer cells were transfected with 10 *μ*g of a FLAG-VP1 expressing plasmid or 10 *μ*g of an empty FLAG vector. The cells were lysed at 30 hpt. Cell lysates were immunoprecipitated with goat anti-STK3 and goat IgG antibodies and subjected to western blotting. The whole-cell lysates and immunoprecipitated antibody-antigen complexes were analyzed by immunoblotting using anti-STK3, anti-FLAG, or anti-*β*-actin antibodies. (d) Similar infection and immunoprecipitation experiments were performed as described above. However, the lysates were immunoprecipitated with mouse anti-FLAG or mouse normal IgG antibodies and subjected to western blotting. (e) HEK293T cells were seeded in Nunc glass bottom dishes, and the monolayer cells were transfected with 1.5 *μ*g of a Myc-STK3 expressing plasmid, 1.5 *μ*g of a FLAG-VP1 expressing plasmid, or 1.5 *μ*g of an empty vector. At 24 hpt, the expression of Myc-STK3 and FLAG-VP1 was detected by an IFA analysis. Cells were double-immunostained for Myc-STK3 (red) and FLAG-VP1 (green); cellular nuclei were counterstained with 4′,6-diamidino-2-phenylindole (DAPI) (blue).

To confirm the interaction of VP1 and STK3, HEK-293T cells were cotransfected with a Myc-STK3 expressing plasmid and a FLAG-VP1 expressing plasmid or an empty FLAG vector. Cell lysates were immunoprecipitated with an anti-Myc antibody and analyzed by western blotting. As shown in Figure 1(a), Myc-STK3 pulled down FLAG-VP1, indicating an interaction between STK3 and VP1. The immunoprecipitated Myc-STK3 protein and the whole-cell lysates were visualized by enhanced chemiluminescence detection reagents. Then, a reverse immunoprecipitation experiment was performed using an anti-FLAG antibody. It was observed that FLAG-VP1 also immunoprecipitated Myc-STK3 (Figure 1(b)).

To further confirm that the FMDV VP1 and STK3 interaction occurs in vivo, PK-15 cells were transfected with a FLAG-VP1 expressing plasmid or an empty FLAG vector. Cell lysates were immunoprecipitated with an anti-STK3 antibody and analyzed by western blotting. As shown in Figure 1(c), STK3 pulled down FLAG-VP1. Then, a reverse immunoprecipitation experiment was performed using an anti-FLAG antibody. Similarly, the results showed that FLAG-VP1 also immunoprecipitated STK3 (Figure 1(d)).

An IFA was performed subsequently, and the results indicated an interaction between VP1 and STK3 (Figure 1(e)). Taken together, these results confirm that FMDV VP1 interacts with STK3.

### 3.2. The Carboxyl-Terminal Region (Amino Acids 180–214) of VP1 Is Essential for the VP1-STK3 Interaction

**Figure 2:**
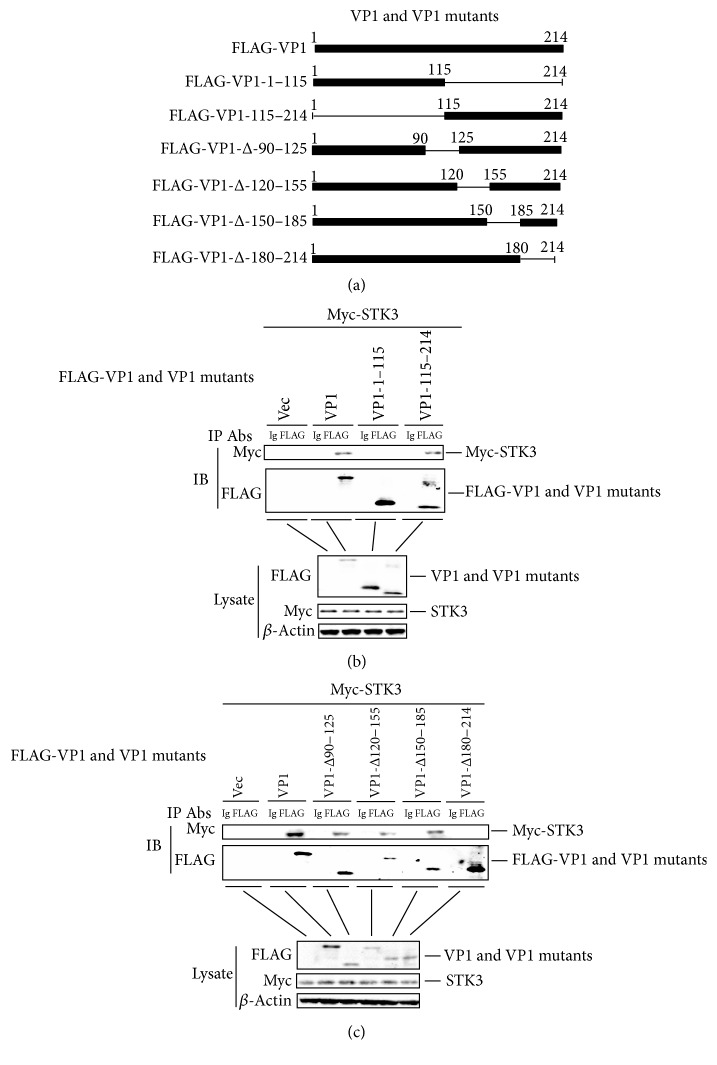
STK3 interacts with VP1 truncated mutants. (a) Schematics showing a series of FLAG-tagged truncated VP1 mutants. (b) HEK-293T cells were seeded in a 10-cm dish, and the monolayer cells were transfected with 8 *μ*g of a Myc-STK3 expressing plasmid, 8 *μ*g of a FLAG-VP1 expressing plasmid, 8 *μ*g of FLAG-VP1 mutants expressing plasmids, or 8 *μ*g of an empty FLAG vector. The cells were lysed at 24 hpt. The lysates were immunoprecipitated with mouse anti-FLAG or mouse IgG antibodies and subjected to western blotting. The immunoprecipitated antibody-antigen complexes and whole-cell lysates were analyzed by anti-FLAG, anti-Myc, or anti-*β*-actin antibodies. (c) Similar transfection and immunoprecipitation experiments were performed as described above.

To identify the structural domains of FMDV VP1 that are responsible for the STK3 interaction, a series of truncated mutants of FMDV VP1 was generated (Figure 2(a)). HEK-293T cells were cotransfected with a Myc-STK3 expressing plasmid and a FLAG-VP1 expressing plasmid, FLAG-VP1 mutants expressing plasmids or an empty FLAG vector. Cell lysates were immunoprecipitated with an anti-FLAG antibody and analyzed by western blotting. The results suggested that the carboxyl-terminal region (amino acids 115–214) of VP1 interacts with STK3 (Figure 2(b)). Next, we examined which amino acids in carboxyl-terminal region of VP1 are required for its interaction with STK3. The results indicated that amino acids 180–214 of FMDV VP1 are essential for the STK3 interaction (Figure 2(c)).

### 3.3. FMDV Infection Decreases Endogenous STK3 Protein Abundance

**Figure 3:**
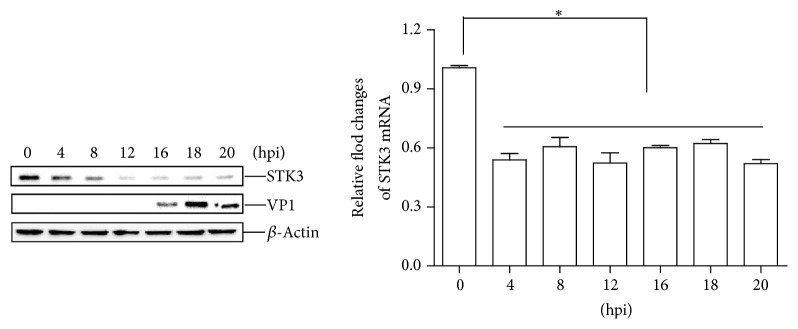
FMDV infection decreases the abundance of the endogenous STK3 protein. PK-15 cells were seeded in 3.5 cm dish, and the monolayer cells were infected with FMDV (MOI 0.5). The cells were collected at the indicated time points (0, 4, 8, 12, 16, 18, and 20 h). The expression of STK3 mRNA was determined by qPCR assay (b). The expression of endogenous STK3 and viral VP1 proteins was determined by western blotting (a).

The abundance of endogenous STK3 during an FMDV infection is unclear. Therefore, PK-15 cells were infected with equal amounts of FMDV at a multiplicity of infection (MOI) of 0.5. The STK3 mRNA level and the STK3 protein abundance were determined and compared over time. The results indicated that both the STK3 mRNA level and STK3 protein abundance decreased at an early infection time (Figure 3). Taken together, we speculate that FMDV reduces the expression of STK3 protein by inhibiting its mRNA level, not at translational level.

### 3.4. STK3 Inhibits FMDV Replication during Virus Infection

**Figure 4:**
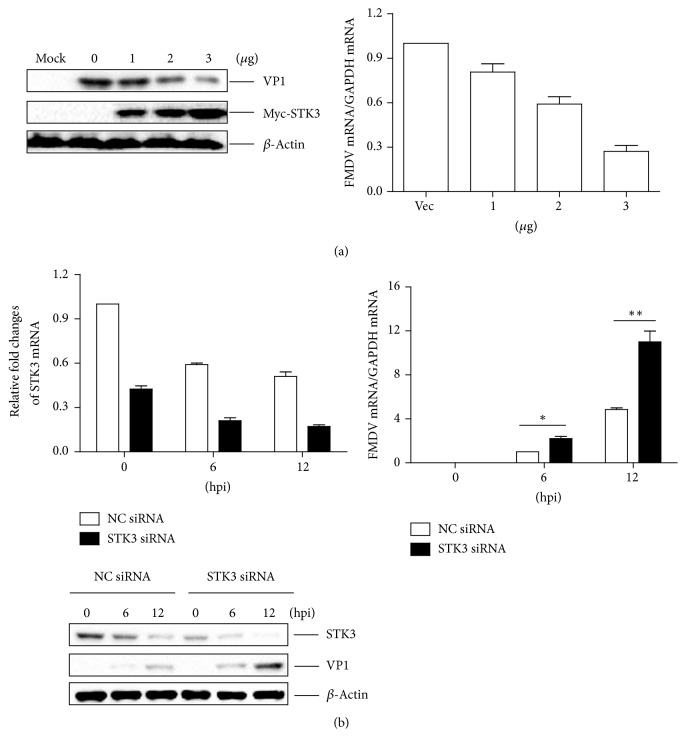
STK3 inhibits FMDV replication during virus infection. (a) PK-15 cells were seeded in 6-well plates, and the monolayer cells were transfected with 1, 2, and 3 *μ*g of a Myc-STK3 expressing plasmid and 3 *μ*g of an empty Myc vector. The empty Myc vector was used in the transfection process to ensure that the same amount of cells received the same amount of total plasmids. At 24 hpt, the cells were mock infected or infected with FMDV (MOI 0.5) for 12 h. The expression of viral RNA and the VP1 protein was detected by qPCR assay and western blotting, respectively. (b) PK-15 cells were seeded in 3.5 cm dish, and the monolayer cells were transfected with 150 nM of STK3 siRNA or NC siRNA for 48 h, followed by infection with equal amounts of FMDV (MOI 0.5). The cells were collected at the indicated time points (0, 6, and 12 h). Expression of STK3 mRNA and viral RNA was determined by qPCR assay. Expression of STK3 and the viral VP1 protein was detected by western blotting.

To assess the role of STK3 in FMDV replication, we evaluated the FMDV yield in PK-15 cells that were transfected with different doses of a Myc-STK3 expressing plasmid. At 24 hpt, the cells were infected with equal amounts of FMDV (MOI 0.5) for 12 h. The FMDV RNA level and protein abundance were compared. As shown in Figure 4(a), overexpression of STK3 significantly suppressed FMDV RNA levels and protein abundance in a dose-dependent manner.

The replication level of FMDV in cells in which STK3 expression was downregulated was further assessed. PK-15 cells were transfected with STK3 siRNA or NC siRNA for 48 h, and then they were infected with equal amounts of FMDV (MOI 0.5). The viral RNA level and the abundance of the VP1 and STK3 proteins in the STK3 siRNA-transfected and NC siRNA-transfected cells were compared at 0, 6, and 12 h after FMDV infection. The results indicated that the FMDV replication level increased significantly in STK3 siRNA-transfected cells (Figure 4(b)).

Taken together, these results demonstrate the important antiviral role of STK3 in FMDV replication.

## 4. Discussion

Viral virulence is dependent on a balanced interaction between viral and cellular proteins. Studies have shown that host protein kinases are important regulators of virus-host interaction and that they play important roles in viral replication [28]. The potential mechanisms by which FMDV proteins interact with host cell proteins are not fully understood. Here, for the first time, we determined that the FMDV structural protein VP1 interacts with porcine STK3 using the yeast two-hybrid system. In concordance with this result, we also demonstrated the interaction of VP1 with STK3 in HEK293T and PK-15 cells using coimmunoprecipitation experiments. Our data also showed that the carboxyl-terminal region (180*–*214) of FMDV VP1 is essential for the interaction with STK3. The G-H loop, containing amino acids 134*–*160 of VP1, plays a significant role in VP1 functions [17]. However, our results indicate that the G-H loop region is not responsible for the interaction of VP1 with STK3.

In humans, studies have indicated that patients lacking STK4, which is a closely related paralog of STK3, showed T- and B-cell lymphopenia and recurrent bacterial and viral infections [29, 30]. STK4, but not STK3, can shut off cytosolic antiviral defense through IRF3 phosphorylation [31]. Besides, downregulation of STK3 expression decreased influenza replication, which indicates that STK3 can facilitate viral replication [32]. In veterinary research, the roles of STK3 in bacterial or viral infections have not been reported. Taken together, the antiviral roles and the exact mechanisms of action of STK3 have not been clarified.

In this study, overexpression and underexpression of STK3 in PK-15 cells were performed. The results confirmed the antiviral role of STK3 in FMDV replication. A recent study showed that Hippo pathway is a potent regulator of cellular antiviral response [33]. STK3 is involved in the Hippo pathway that activates various functions [19]. Therefore, we speculate that STK3 inhibits FMDV replication via the Hippo pathway. Determining the specific antiviral mechanisms mediated by the Hippo pathway requires further studies.

In conclusion, our results showed, for the first time, that the porcine cellular protein STK3 interacts with FMDV VP1. The VP1-STK3 interaction may be critical for modulating viral replication. The results also described a novel antiviral role of STK3 during FMDV infection. This presents possible new VP1 functions in viral pathogenesis, and further studies need to be performed to identify other cellular proteins that may interact with FMDV proteins, which will promote a better understanding of FMDV pathogenesis.

## Figures and Tables

**Table 1:** qPCR primers used in this study.

Primers	Sequences (5′-3′)	Use
STK3-F	TTTTGGATGGCTCCTGAGGTAAT	Detection of porcine STK3 gene
STK3-R	TGAATGTTGGTGGTGGGTTTGTG	
FMDV-F	CACTGGTGACAGGCTAAGG	Detection of FMDV gene
FMDV-R	CCCTTCTCAGATTCCGAGT	
GAPDH-F	ACATGGCCTCCAAGGAGTAAGA	Detection of porcine GAPDH gene
GAPDH-R	GATCGAGTTGGGGCTGTGACT	
